# Association between FGF23, α-Klotho, and Cardiac Abnormalities among Patients with Various Chronic Kidney Disease Stages

**DOI:** 10.1371/journal.pone.0156860

**Published:** 2016-07-11

**Authors:** Suguru Tanaka, Shu-ichi Fujita, Shun Kizawa, Hideaki Morita, Nobukazu Ishizaka

**Affiliations:** Department of Cardiology, Osaka Medical College, Osaka, Japan; The University of Tokyo, JAPAN

## Abstract

**Background:**

Several experimental studies have demonstrated that fibroblast growth factor 23 (FGF23) may induce myocardial hypertrophy via pathways independent of α-Klotho, its co-factor in the induction of phosphaturia. On the other hand, few studies have clearly demonstrated the relationship between FGF23 level and left ventricular hypertrophy among subjects without chronic kidney disease (CKD; i.e., CKD stage G1 or G2).

**Purpose:**

To investigate the data from 903 patients admitted to the cardiology department with various degrees of renal function, including 234 patients with CKD stage G1/G2.

**Methods and Results:**

Serum levels of full-length FGF23 and α-Klotho were determined by enzyme immunoassay. After adjustment for sex, age, and estimated glomerular filtration rate (eGFR), the highest FGF23 tertile was significantly associated with left ventricular hypertrophy among patients with CKD stage G1/G2 and those with CKD stage G3a/G3b/G4 as compared with the lowest FGF23 tertile, and the association retained significance after further adjustment for serum levels of corrected calcium, inorganic phosphate, and C-reactive protein, as well as diuretic use, history of hypertension, and systolic blood pressure. FGF23 was also associated with low left ventricular ejection fraction among patients with CKD stage G1/G2 and those with CKD stage G3a/G3b/G4 after adjusting for age, sex, eGFR, corrected calcium, and inorganic phosphate. On the other hand, compared with the highest α-Klotho tertile, the lowest α-Klotho tertile was associated with left ventricular hypertrophy and systolic dysfunction only among patients with CKD stage G3b and stage G3a, respectively.

**Conclusions:**

An association between FGF23 and cardiac hypertrophy and systolic dysfunction was observed among patients without CKD as well as those with CKD after multivariate adjustment. However, the association between α-Klotho and cardiac hypertrophy and systolic dysfunction was significant only among patients with CKD G3b and G3a, respectively.

## Introduction

Fibroblast growth factor 23 (FGF23) is a bone-secreted circulating endocrine hormone that causes phosphaturic effects [1] via the formation of heterodimeric complexes consisting of FGF receptors and the specific FGF23 co-receptor, α-Klotho [2,3], which was first identified as a protein with anti-aging properties [4]. Although the precise mechanisms remain unclear, serum FGF23 levels increase with a decline of renal function leading to reduced excretion of urinary phosphate [5,6].

In addition to these effects on maintaining phosphate homeostasis, several studies have shown an association between FGF23 and cardiac hypertrophy and/or left ventricular dysfunction in various populations such as patients with chronic kidney disease (CKD) [7,8], elderly individuals [9], and those undergoing maintenance hemodialysis [10,11]. A possible association between circulating α-Klotho and cardiovascular disease has also been demonstrated in clinical studies [12,13].

Experimental studies have suggested the direct cardiac effects of FGF23 and α-Klotho; for example, intramyocardial injection of FGF23 ameliorated the development of cardiac hypertrophy [7], and cardiac hypertrophy induced by certain pathologic conditions was found to be exaggerated in heterozygous Klotho-deficient mice and was lessened by either transfer of the *klotho* gene [14] or treatment with Klotho protein [15]. These studies indicate that FGF23 and α-Klotho may not be merely bystanders of cardiac abnormalities, but rather may directly aggravate or ameliorate cardiac injury.

Most epidemiological studies assessing the relationship between circulating levels of FGF23 or α-Klotho and cardiac abnormalities have been performed among a population that either exclusively has renal dysfunction or includes many such subjects. According to the above-mentioned experimental studies, FGF23 and/or α-Klotho may induce or reduce cardiac hypertrophy; however, clinical data demonstrating an association between circulating levels of FGF23 and/or α-Klotho and cardiac abnormalities among subjects without renal dysfunction remain limited. In our previous study, we demonstrated that serum FGF23 levels were positively and negatively associated with, respectively, left ventricular hypertrophy (LVH) and systolic dysfunction among cardiology inpatients; owing to the relatively small population, however, these associations could not be statistically assessed according to CKD stage [16]. To this end, we herein investigated whether FGF23 is associated with cardiac hypertrophy and systolic dysfunction by analyzing data from total of 903 patients with various stages of CKD.

## Methods

### Ethics Statement

Written informed consent was obtained from all patients or their guardians. The current retrospective study was approved by the Ethics Committee at the Osaka Medical College and conducted in accordance with the Declaration of Helsinki.

### Study Population

Data from 903 consecutive patients with sufficient clinical data for the current analysis who were admitted to the cardiology department between January 2012 and June 2014 and gave informed consent were assessed in this study. For each study patient, a history of hypertension, diabetes, and smoking status was determined from the clinical record.

### Laboratory Analysis

Blood samples were collected in the morning after an overnight fast. Aliquots of serum and plasma were immediately obtained and stored at -80 degrees until analysis. Serum levels of α-Klotho were measured by using a solid-phase sandwich enzyme-linked immunosorbent assay (ELISA) (Immuno-Biological Laboratories, Gunma, Japan) [17]. Serum levels of intact FGF23 were measured by using a two-step FGF23 enzyme immunoassay (ELISA) kit (Kainos Laboratories Inc., Tokyo, Japan) according to the manufacturer’s instructions. Calcium, inorganic phosphate, and C-reactive protein (CRP) were measured by routine laboratory methods. When serum albumin was 4 mg/dL or lower, serum calcium levels were corrected by the formula [calcium + (4–“serum albumin”)], and designated as corrected calcium. Serum levels of intact parathyroid hormone (PTH) were measured by the immunochemical detection method (LSI Medience, Tokyo, Japan).

### Determination of CKD stage

The estimated glomerular filtration rate (eGFR) was calculated by the following Modification of Diet in Renal Disease equation for Japanese subjects: eGFR (mL/min/1.73 m^2^) = 194 × (serum creatinine)^-1.094^ × (age)^-0.287^ (× 0.739, when female) [18]. Renal function was graded as CKD stage G1 to G5 on the basis of eGFR level or requirement for hemodialysis [19], and the G3 category was further subdivided into early stage (G3a) and late stage (G3b) [20] as follows: G1 (eGFR > 90 mL/min/m^2^); G2 (eGFR 60–89 mL/min/m^2^); G3a (eGFR 45–59 mL/min/m^2^); G3b (eGFR 30–44 mL/min/m^2^); G4 (eGFR 15–29 mL/min/m^2^); and G5 (eGFR <15 mL/min/m^2^). For analysis, we combined patients with CKD G1 and G2 (stage G1/G2) (i.e., subjects with eGFR of ≥60 mL/min/1.73m^2^) in one group as “no-CKD”.

### Echocardiography

Echocardiographic examinations were performed with a Vivid 7 Dimension equipped with a multi-frequency transducer (GE Healthcare, Vingmed, Norway). Left ventricular (LV) end-diastolic dimension (LVDd), interventricular septal thickness (IVST) and posterior wall thickness (PWT) were measured at end diastole. LV volumes were calculated by the modified Simpson method using the apical 4-chamber view. The LV ejection fraction (LVEF) was defined as low when < 50%. For calculation of the LV mass (LVM), we used the formula proposed by Devereux et al. [21] with modification as follows: 0.8 x 1.04 x [(LVDd + IVST + PWT)^3^—LVDd^3^] + 0.6 [22]. Body surface area (BSA) was calculated by using the following formula: (body weight)^0.425^ × (height)^0.725^ × 0.007184, and the LVM index (LVMI) was calculated as the ratio of LVM to BSA. When the LVMI was greater than 118 g/m^2^ (men) or 108 g/m^2^ (women), LV hypertrophy was defined as present [23].

### Statistical Analysis

Baseline characteristics were assessed with standard descriptive statistics. Data were expressed either as mean ± standard deviation (for parameters normally distributed) or median and interquartile range (IQR) (for parameters not normally distributed). Spearman rank correlation test was used to assess the correlation between two variables that were not normally distributed. Data analysis was performed by SPSS statistics version 21.0 (IBM, Armonk, NY). Multivariate receiver operating characteristic (ROC) curve analysis was performed by STATA 12 (StataCorp LP, College Station, TX).

## Results

### Patient Characteristics

**Fig 1:**
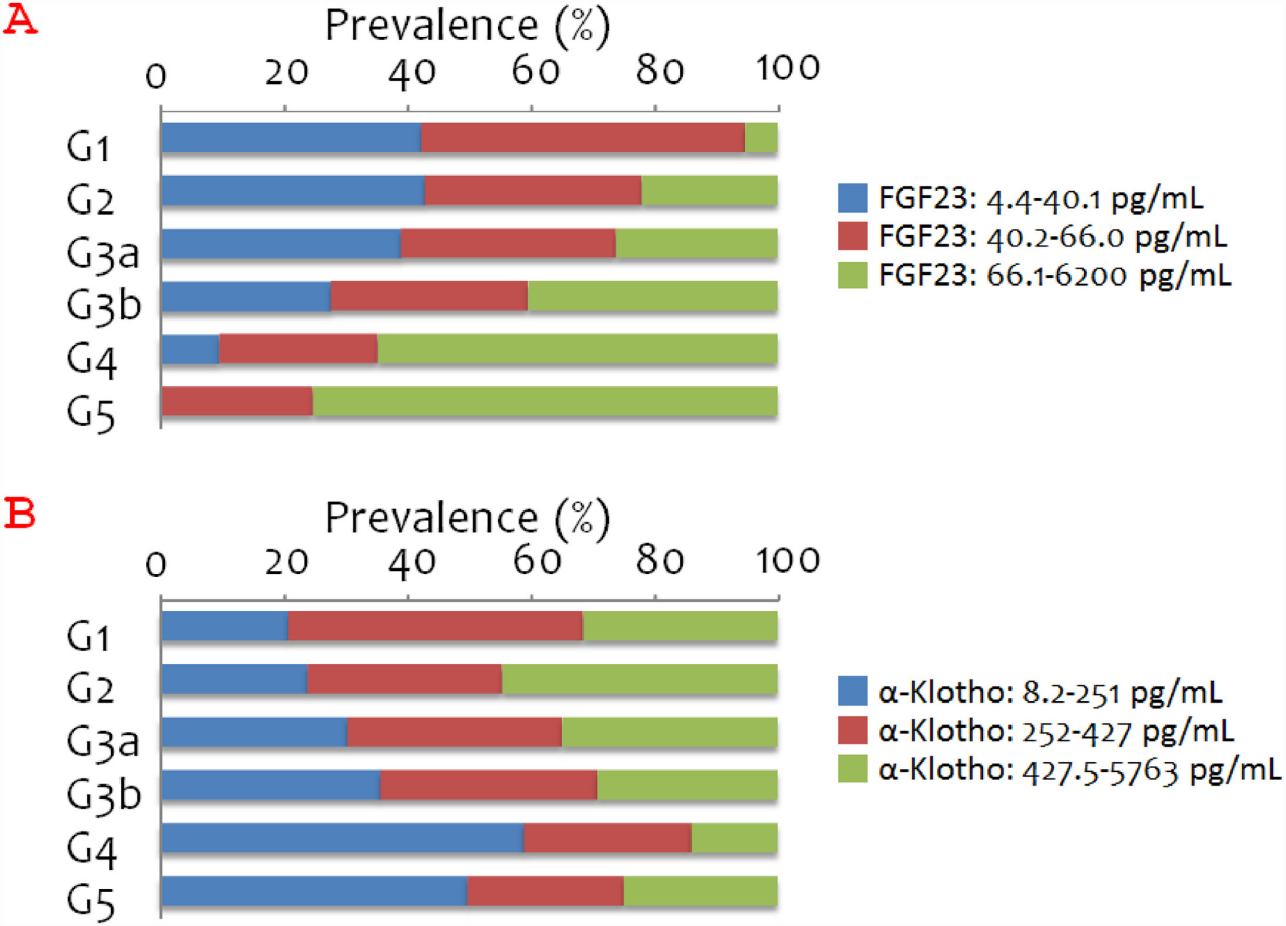
Prevalence of each FGF23 or α-Klotho tertile by CKD stage. A. Percentage of patients in each FGF23 tertile according to CKD stage (P<0.001 by χ^2^ test). B. Percentage of patients in each α-Klotho tertile according to CKD stage (P<0.001 by *χ*^2^ test).

Of the 903 patients enrolled, 19 (2.1%), 215 (23.8%), 311, (34.4%), 253 (28.0%), and 93 (10.3%), and 12 (1.3%) had CKD stage G1, G2, G3a, G3b, G4, and G5, respectively. Thus, about a quarter of the study population (234 patients, 25.9%) were considered to have no-CKD. Ischemic heart disease and arrhythmic disease were, respectively, the first and the second most common disorders among the study subjects. A history of hypertension was more prevalent among patients with CKD (stages G3a, G3b, G4, and G5) than among patients without CKD (stage G1/G2), although systolic blood pressure did not differ significantly across the groups (Table 1). Among the overall population, 259 (28.7%) patients were taking diuretic drug(s) (thiazide and/or loop diuretic), and serum FGF23 was significantly higher among these patients (median, 64.0 pg/mL; IQR, 45.1–104.4 pg/mL) than among the remaining 644 patients without diuretic use (median, 46.5 pg/mL; IQR, 32.2–69.4 pg/mL; P<0.001 by Mann-Whitney test). CRP values were found to increase with advancing CKD stage (Table 2). FGF23 concentrations also increased with advancing CKD stage (Fig 1A).

**Table 1:** Demographic characteristics of the study patients.

	CKD stage					P Value
Variables	G1/G2	G3a	G3b	G4	G5	
eGFR range	≥60	45–59	30–44	15–30	<15	
Number of patients	234	311	253	93	12	
Women/men	127 / 107	57 / 254	38 / 215	15 / 78	1 / 11	<0.001
Age, years	63.3 ± 13.2	67.8 ± 10.4	72.3 ± 8.9	76.5 ± 6.1	66.7 ± 9.6	<0.001
Body mass index, kg/m^2^	22.8 ± 3.8	23.7 ± 3.4	23.5 ± 3.4	23.1 ± 2.7	23.9 ± 2.9	0.020
Systolic blood pressure, mmHg	127 ± 18	128 ± 19	125 ± 20	125 ± 21	135 ± 26	0.373
Pulse rate, bpm	73.8 ± 15.5	73.5 ± 18.2	74.1 ± 17.4	73.0 ± 18.0	78.7 ± 19.9	0.853
*Smoking status*						
Never, n (%)	127 (54.3)	96 (30.9)	80 (31.6)	29 (31.2)	3 (25.0)	0.744
Former, n (%)	78 (33.3)	167 (53.7)	143 (56.5)	59 (63.4)	6 (50.0)	
Current, n (%)	29 (12.4)	48 (15.4)	30 (11.9)	5 (5.4)	3 (25.0)	
Hypertension	150 (64.1)	224 (72.0)	200 (79.1)	68 (73.1)	9 (75.0)	0.009
Diabetes	60 (25.6)	90 (28.9)	86 (34.0)	39 (41.9)	5 (41.7)	0.031
*Cardiovascular disease*						
Ischemic heart disease, n (%)	146 (62.4)	220 (70.7)	185 (73.1)	61 (65.6)	11 (91.7)	0.031
Arrhythmic disease, n (%)	79 (33.8)	90 (28.9)	68 (26.9)	23 (24.7)	0 (0.0)	0.064
Peripheral artery disease, n (%)	11 (4.7)	22 (7.1)	23 (9.1)	11 (11.8)	2 (16.7)	0.114
Valvular heart disease, n (%)	10 (4.3)	29 (9.3)	21 (8.3)	11 (11.8)	2 (16.7)	0.085
Cardiomyopathy, n (%)	19 (8.1)	15 (4.8)	24 (9.5)	14 (15.1)	1 (8.3)	0.024
Aneurysmal disease, n (%)	7 (3.0)	9 (2.9)	15 (5.9)	5 (5.4)	0 (0.0)	0.284
*Medication*						
ACE inhibitors/ARB, n (%)	94 (40.2)	165 (53.1)	159 (62.8)	54 (58.1)	6 (50.0)	<0.001
Beta blockers, n (%)	76 (32.5)	128 (41.2)	103 (40.7)	39 (41.9)	8 (66.7)	0.055
Calcium channel blockers, n (%)	94 (40.2)	131 (42.1)	118 (46.6)	39 (41.9)	6 (50.0)	0.642
Diabetic medication, n (%)	55 (23.5)	69 (22.2)	75 (29.6)	29 (31.2)	5 (41.7)	0.103
Statin, n (%)	100 (42.7)	164 (52.7)	135 (53.4)	44 (47.3)	7 (58.3)	0.107
Loop, n (%)	29 (12.4)	67 (21.5)	78 (30.8)	50 (53.8)	2 (16.7)	<0.001
Thiazide, n (%)	11 (4.7)	11 (3.5)	14 (5.5)	9 (9.7)	0 (0.0)	0.163
Aldosterone antagonist, n (%)	9 (3.8)	25 (8.0)	44 (17.4)	10 (10.8)	1 (8.3)	<0.001

ACE, angiotensin converting enzyme; ARB, angiotensin receptor blocker.

**Table 2:** Laboratory and echocardiographic data of the study patients.

	CKD stage					
Variables	G1/G 2	G3a	G3b	G4	G5	P value
Number of patients	234	311	253	93	12	
White blood cell count, x10^3^/mL	5.62 (4.73–6.84)	5.77 (4.85–7.09)	5.97 (4.96–7.09)	5.93 (4.77–7.15)	6.31 (4.83–6.93)	0.434
Hemoglobin, g/dL	13.5 (12.5–14.5)	13.8 (12.7–14.9)	13.1 (11.9–14.3)	11.3 (10.1–13.3)	12.8 (10.2–14.5)	<0.001
Platelet count, x10^4^/mL	22.4 (18.5–26.4)	20.6 (18.2–24.8)	19.5 (16.5–22.6)	18.4 (13.3–24.0)	16.5 (12.5–20.8)	<0.001
Total protein, g/dL	7.0 (6.7–7.4)	7.0 (6.7–7.4)	6.9 (6.6–7.4)	6.9 (6.6–7.2)	7.1 (6.6–7.6)	0.700
Albumin, g/dL	4.1 (3.8–4.3)	4.1 (3.8–4.3)	3.9 (3.6–4.1)	3.7 (3.5–4.0)	3.9 (3.4–4.3)	<0.001
Blood urea nitrogen, mg/dL	14 (12–16)	16 (14–19)	19 (17–23)	31 (24–43)	27 (19–43)	<0.001
Serum creatinine, mg/dL	0.66 (0.60–0.70)	0.84 (0.78–0.90)	1.09 (1.01–1.19)	1.69 (1.48–2.05)	5.14 (0.97–7.83)	<0.001
eGFR, mL/min/1.73m2	71.7±10.5	52.3±4.3	38.2±4.1	22.6±5.5		<0.001
C-reactive protein, mg/dL	0.08 (0.03–0.22)	0.08 (0.04–0.27)	0.13 (0.05–0.37)	0.22 (0.08–0.80)	0.29 (0.03–0.67)	<0.001
FGF23, pg/mL	45 (32–63)	46 (32–67)	59 (37–81)	85 (60–130)	264 (68–2500)	<0.001
α-Klotho, pg/mL	388 (254–532)	337 (217–489)	316 (199–464)	215 (135–331)	249 (100–452)	<0.001
Corrected calcium, mg/dL	9.1(8.9–9.4)	9.1(9–9.4)	9.2(8.9–9.4)	9.1(9–9.6)	9.3(9–9.475)	0.924
Inorganic phosphate, mg/dL	3.5 (3.1–3.8)	3.3 (3.0–3.7)	3.3 (3.0–3.7)	3.3 (3.0–3.9)	4.0 (3.4–5.1)	0.001
iPTH (n = 169/222/181/61/11)	32 (22–41)	34 (24–44)	34 (23–45)	46 (31–71)	27 (23–41)	<0.001
Echocardiographic data						
LVDd, mm	9.1(8.9–9.4)	9.1(9–9.4)	9.2(8.9–9.4)	9.1(9–9.6)	9.3(9–9.475)	0.924
LVEF, %	63 (56–68)	61 (54–67)	58 (48–66)	57 (42–64)	57 (39–62)	0.023
LVMI, g/cm^2^	32 (22–41)	34 (24–44)	34 (23–45)	46 (31–71)	27 (23–41)	<0.001

LVDd, left ventricular diastolic dimension; LVEF, left ventricular ejection fraction; LVMI, left ventricular mass index.

The proportion of patients in the highest FGF23 tertile increased with declining renal function (Fig 1A). Similarly, the proportion of patients in the lowest α-Klotho tertile increased with declining renal function, although the proportion of patients in the lowest α-Klotho tertile was greater among patients with CKD stage G4 (59%) than among those with CKD stage G5 (50%) (Fig 1B).

The correlation coefficient for the association between FGF23 and α-Klotho was 0.81 (P = 0.743), 0.12 (P = 0.837), -0.12 (P = 0.080), -0.03, (P = 0.670), 0.11 (P = 0.304), and -0.24 (P = 0.443) among patients who had CKD stage G1, G2, G3a, G3b, G4, and G5, respectively.

**Fig 2:**
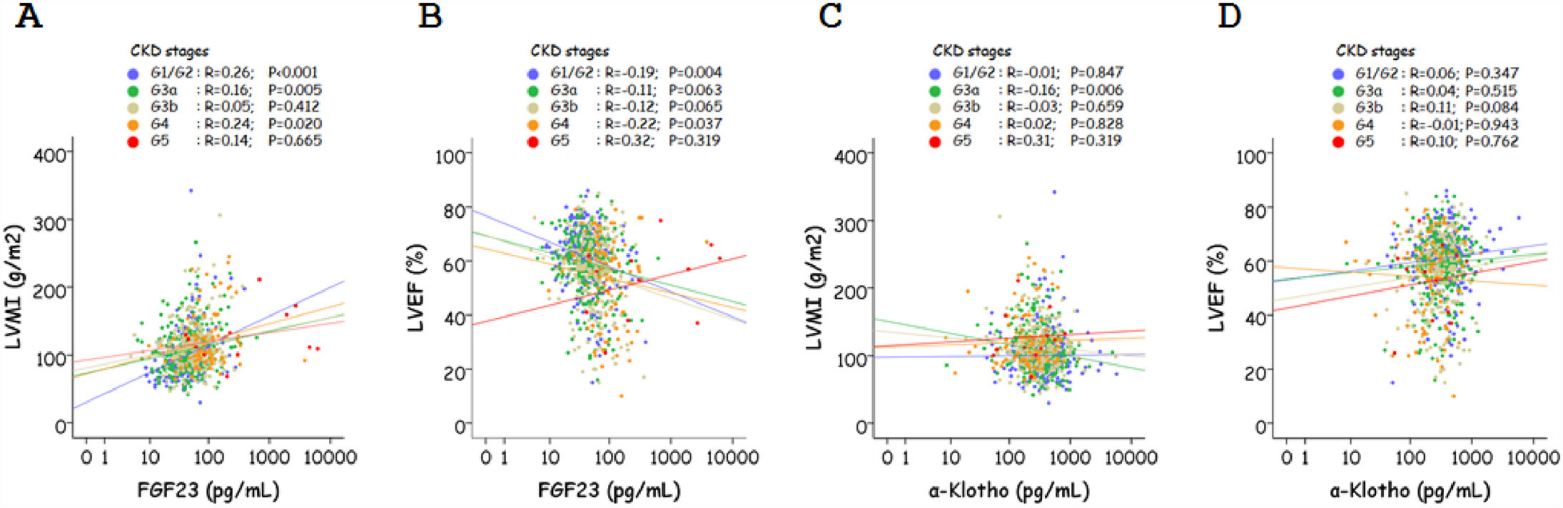
Correlation between FGF23, α-Klotho, left ventricular mass index (LVMI), and left ventricular ejection fraction (LVEF) according to CKD stages. A. Correlation between FGF23 and LVMI. B. Correlation between FGF23 and LVEF. C. Correlation between α-Klotho and LVMI. D. Correlation between α-Klotho and LVEF. The results of Spearman’s correlation test for each CKD stage subgroup are shown.

Among the various CKD stage subgroups (G1/G2, G3a, G3b, G4, G5), the correlation between FGF23 and LVMI was significant among those with CKD stage G1/G2, G3a, and G4 (Fig 2A) and that between FGF23 and LVEF was significant among those with CKD stage G1/G2, and G4 (Fig 2B). On the other hand, the correlation between α-Klotho and LVMI was significant among those with CKD stage G3a (Fig 2C), but that between α-Klotho and LVEF did not reach statistical significance among any of the CKD stage subgroups (Fig 2D).

### Association between tertiles of FGF23/α-Klotho and LVEF/LVMI among patients with various stages of CKD

**Fig 3:**
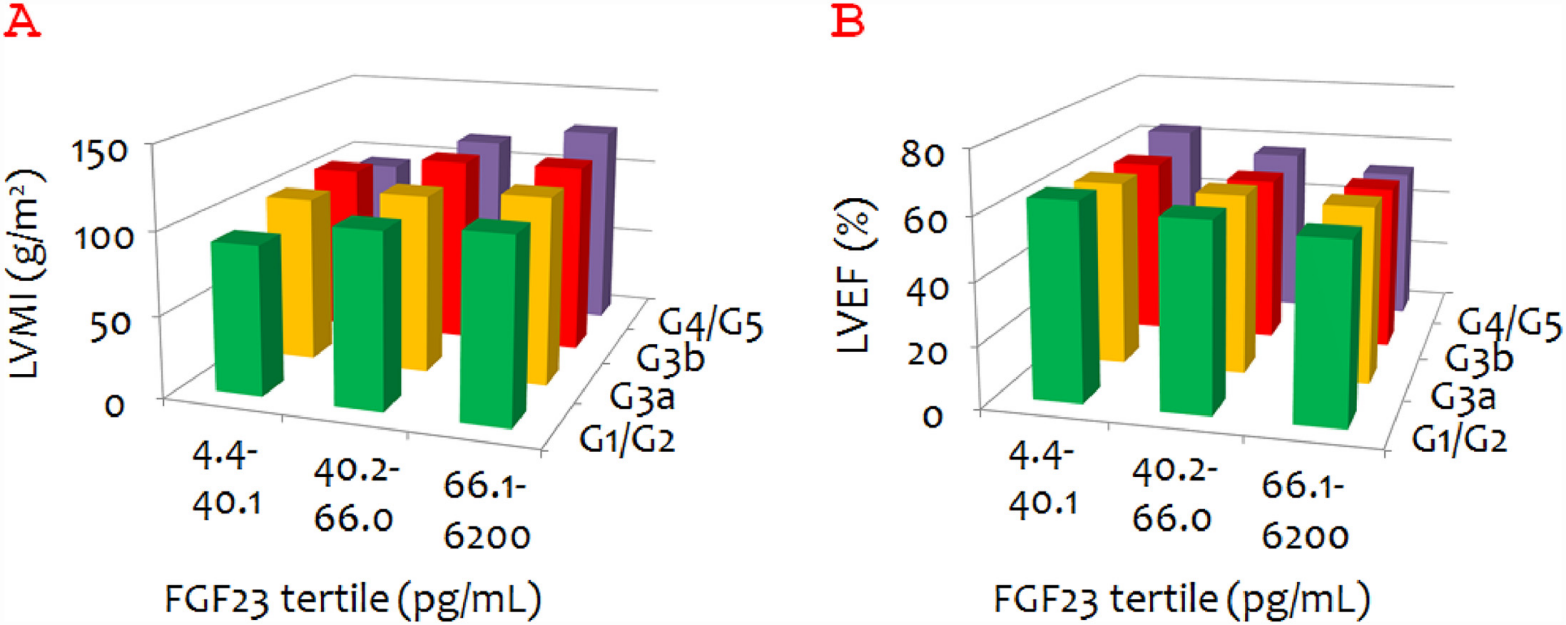
Mean left ventricular mass index (LVMI) and left ventricular ejection fraction (LVEF) according to FGF23 tertile and chronic kidney disease (CKD) stage. A. Mean LVMI according to FGF23 tertile and CKD stage. B. Mean LVEF according to FGF23 tertile and CKD stage.

When the data were assessed as tertiles of FGF23 by CKD stage, the mean LVMI seem to increase with FGF23 value in all CKD stage subgroups (Fig 3A). In fact, LVMI differed significantly across the FGF23 tertiles among patients with CKD stages G1/G2 (P = 0.002, by Kruskal-Wallis analysis), G3a (P = 0.013), and G4/G5 (P = 0.035). Similarly, mean LVEF seemed to decrease with FGF23 value irrespective of CKD stage (Fig 3B). LVEF significantly differed across the FGF23 tertiles among patients with CKD stages G1/G2 (P = 0.014) and G4/G5 (P = 0.039).

**Fig 4:**
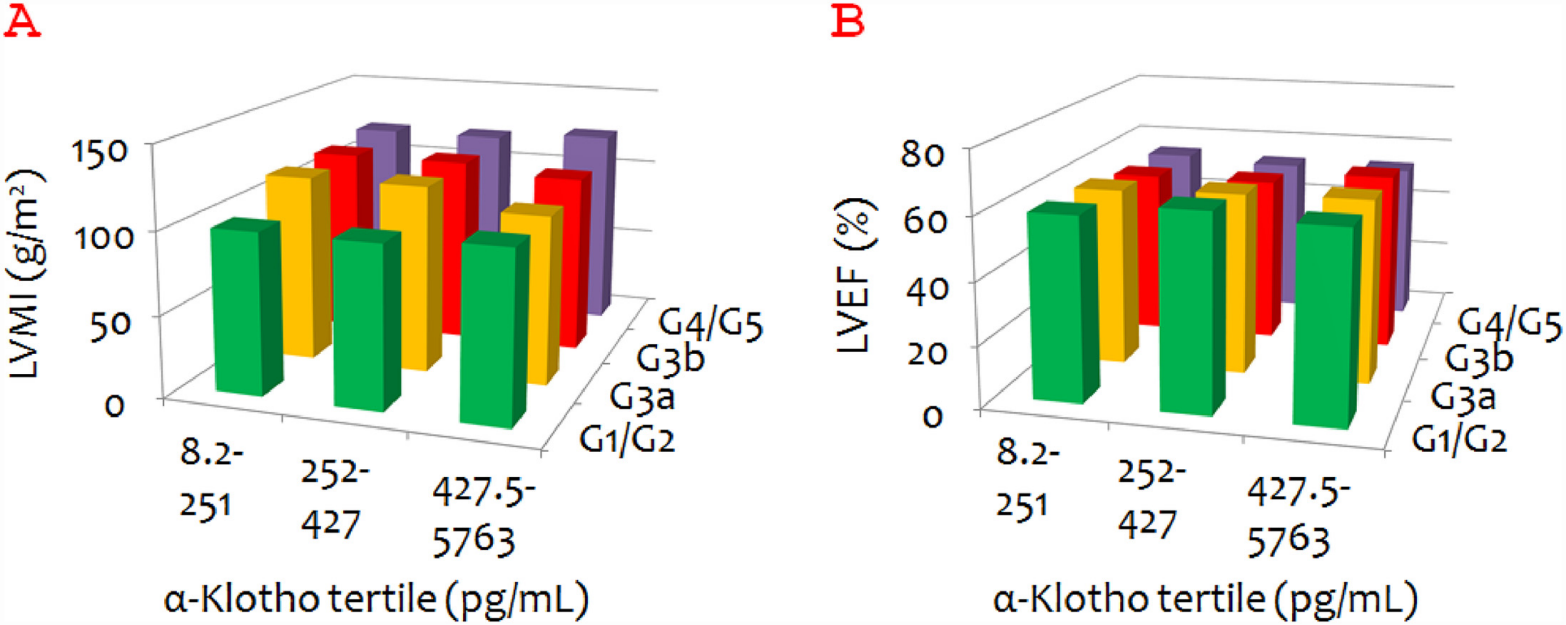
Mean left ventricular mass index (LVMI) and left ventricular ejection fraction (LVEF) according to α-Klotho tertile and chronic kidney disease (CKD) stage. A. Mean LVMI according to α-Klotho tertile and CKD stage. B. Mean LVEF according to α-Klotho tertile and CKD stage.

Unlike FGF23, α-Klotho did not seem to be associated with either LVMI or LVEF (Fig 4A and 4B). α-Klotho was found to be negatively associated with LVMI only among patients with CKD stage G3a (P = 0.042).

### Multivariate logistic regression analysis for the association between FGF23 and LVH and low LVEF

We first examined the univariate association of various parameters with LVH or low LVEF; including age, BMI, systolic blood pressure, pulse rate, white blood cell count, hemoglobin, platelet count, total protein, albumin, uric acid, CRP, eGFR, cCa, iP, FGF23, α-Klotho (by Mann-Whitney test), sex, smoking status, use of either diuretics (loop or thiazide), statin, angiotensin converting enzyme (ACE)/angiotensin receptor blocker (ARB), β blocker, calcium channel blocker, and aldosterone antagonist (by *χ*^2^ test). Among these, a significant association with LVH was found for hemoglobin (P = 0.001), albumin (P < 0.001), uric acid (P = 0.002), CRP (P = 0.009), eGFR (P = 0.001), iPTH (P = 0.032), FGF23 (P < 0.001), α-Klotho (P = 0.016), use of diuretic (P < 0.001), ACE/ARB (P = 0.009) β blocker (P < 0.001), aldosterone antagonist (P < 0.001); and a significant association with low LVEF was found for systolic blood pressure (P < 0.001), hemoglobin (P = 0.001), platelet count (P = 0.002), albumin (P < 0.001), uric acid (P < 0.001), CRP (P < 0.001), eGFR (P < 0.001), iPTH (P = 0.036), FGF23 (P < 0.001), α-Klotho (P = 0.007), use of diuretic (P < 0.001), β blocker (P < 0.001), and aldosterone antagonist (P < 0.001)

In multivariate logistic regression analysis, as compared with the lowest FGF23 tertile, both the middle and the highest FGF23 tertiles were significantly associated with LVH among patients with CKD stage G1/G2 and those with stage G3a after adjustment for sex, age, and eGFR (Table 3, model 1). This association remained significant after further adjustment for serum levels of corrected calcium, inorganic phosphate (model 2), CRP, diuretic use model 3), and intact PTH (model 5). After adjustment for sex, age, and eGFR, corrected calcium, inorganic phosphate CRP, diuretic use model, systolic blood pressure, and history of hypertension (model 4), the association between the highest FGF23 tertile and LVH Association between the highest FGF23 and LVH was found to be borderline significant (P = 0.05).

**Table 3:** Logistic regression analysis for the association between FGF23 and left ventricular mass or left ventricular ejection fraction.

		FGF23 t1		FGF23 t2			FGF23 t3		
		OR		OR	95% CI		OR	95% CI	
**Dependent variable: LVH**									
*model 1*	G1/G2	1	(ref)	1.66	(0.83–3.32)		2.85	(1.30–6.25)	**
	G3a	1	(ref)	1.22	(0.69–2.15)		2.03	(1.11–3.71)	*
	G3b	1	(ref)	1.57	(0.78–3.17)		1.70	(0.87–3.30)	
	G4	1	(ref)	10.7	(1.03–112)	*	12.6	(1.29–123)	*
*model 2*	G1/G2	1	(ref)	1.66	(0.83–3.31)		2.82	(1.28–6.19)	*
	G3a	1	(ref)	1.29	(0.72–2.32)		2.50	(1.33–4.71)	**
	G3b	1	(ref)	1.69	(0.83–3.45)		1.83	(0.93–3.62)	
	G4	1	(ref)	13.6	(1.24–149)	*	17.6	(1.66–185)	*
*model 3*	G1/G2	1	(ref)	1.54	(0.75–3.17)		2.67	(1.16–6.18)	*
	G3a	1	(ref)	1.25	(0.69–2.27)		2.39	(1.25–4.56)	**
	G3b	1	(ref)	1.7	(0.79–3.41)		1.68	(0.84–3.36)	
	G4	1	(ref)	13.0	(1.18–143)	*	16.1	(1.52–171)	*
*model 4*	G1/G2	1	(ref)	1.40	(0.67–2.90)		2.36	(1.00–5.58)	
	G3a	1	(ref)	1.23	(0.67–2.26)		2.37	(1.23–4.56)	*
	G3b	1	(ref)	1.63	(0.78–3.40)		1.69	(0.84–3.40)	
	G4	1	(ref)	13.2	(1.13–154)	*	16.65	(1.51–184)	*
*model 5*	G1/G2	1	(ref)	1.48	(0.61–3.60)		2.84	(1.05–7.64)	*
	G3a	1	(ref)	1.85	(0.89–3.84)		3.01	(1.37–6.61)	**
	G3b	1	(ref)	1.88	(0.72–4.90)		1.67	(0.69–4.03)	
	G4	1	(ref)	15.0	(0.89–254)		14.50	(0.88–240)	
**Dependent variable: Low LVEF**									
*model 1*	G1/G2	1	(ref)	1.30	(0.49–3.42)		3.44	(1.24–9.59)	*
	G3a	1	(ref)	2.19	(0.97–4.93)		3.19	(1.37–7.41)	**
	G3b	1	(ref)	1.69	(0.77–3.72)		1.85	(0.87–3.92)	
	G4	1	(ref)	2.93	(0.30–29.1)		5.23	(0.59–46.1)	
*model 2*	G1/G2	1	(ref)	1.21	(0.45–3.22)		3.25	(1.16–9.13)	*
	G3a	1	(ref)	2.11	(0.93–4.81)		3.29	(1.38–7.86)	**
	G3b	1	(ref)	1.63	(0.73–3.62)		1.78	(0.83–3.82)	
	G4	1	(ref)	3.51	(0.35–35.6)		6.48	(0.71–59.1)	
*model 3*	G1/G2	1	(ref)	0.87	(0.30–2.49)		2.60	(0.85–7.90)	
	G3a	1	(ref)	1.92	(0.81–4.58)		3.17	(1.27–7.92)	*
	G3b	1	(ref)	1.40	(0.57–3.45)		1.34	(0.57–3.19)	
	G4	1	(ref)	2.48	(0.21–28.9)		4.59	(0.46–46.4)	
*model 4*	G1/G2	1	(ref)	0.85	(0.29–2.46)		2.57	(0.82–8.11)	
	G3a	1	(ref)	2.06	(0.86–4.94)		3.49	(1.39–8.80)	**
	G3b	1	(ref)	1.46	(0.59–3.62)		1.39	(0.58–3.30)	
	G4	1	(ref)	3.21	(0.25–41.1)		5.81	(0.52–64.4)	
*model 5*	G1/G2	1	(ref)	0.70	(0.17–2.85)		3.64	(0.87–15.30)	
	G3a	1	(ref)	1.15	(0.39–3.34)		2.71	(0.95–7.73)	
	G3b	1	(ref)	1.39	(0.42–4.62)		1.18	(0.39–3.61)	
	G4	1	(ref)	0.68	(0.03–13.6)		1.44	(0.10–20.0)	

Model 1, adjusted for sex, age and eGFR; model 2, adjusted for variables in model 1 plus corrected calcium and inorganic phosphate; model 3, adjusted for variables used in model 2 plus CRP, and diuretic use; model 4, adjusted for variables used in model 3 plus systolic blood pressure and history of hypertension; model 5, adjusted for variables used in model 3 plus intact PTH, although fewer patients were included in this analysis.

* and ** indicate p<0.05 and P<0.01, respectively;

t1, t2, and t3 indicate the lowest, middle, and highest tertile, respectively. OR indicates odds ratio, and ref indicates referent.

The association between the highest FGF23 tertile and low LVEF was also found to be significant after adjustment for age, sex, eGFR, (model 1); and corrected calcium, and inorganic phosphate (model 2); however, it lost statistical significance after further adjustment for diuretic use and CRP (models 3, 4, 5).

### Multivariate logistic regression analysis for the association between α-Klotho and LVH and low LVEF

As compared with the highest α-Klotho tertile, the lowest α-Klotho tertile was not associated with LVH or low LVEF among patients with CKD stage G1/G2 irrespective of the model used (Table 4). On the other hand, the lowest α-Klotho tertile was significantly positively associated with LVH among patients with CKD stage G3a, and with low LVEF among those with CKD stage G3b, whichever model was used.

**Table 4:** Logistic regression analysis for the association between α-Klotho and left ventricular mass or left ventricular ejection fraction.

		α-Klotho t1			α-Klotho t2		α-Klotho t3	
		OR	95% CI		OR	95% CI	OR	
**Dependent variable: LVH**								
*model 1*	G1/G2	1.10	(0.54–2.25)		0.66	(0.33–1.34)	1	(ref)
	G3a	2.18	(1.21–3.95)	**	1.27	(0.70–2.32)	1	(ref)
	G3b	1.27	(0.65–2.47)		1.31	(0.68–2.53)	1	(ref)
	G4	1.46	(0.39–5.46)		1.7	(0.41–7.34)	1	(ref)
*model 2*	G1/G2	1.12	(0.55–2.29)		0.67	(0.33–1.35)	1	(ref)
	G3a	2.24	(1.23–4.09)	**	1.25	(0.68–2.28)	1	(ref)
	G3b	1.30	(0.67–2.53)		1.33	(0.69–2.57)	1	(ref)
	G4	1.98	(0.45–8.70)		2.60	(0.52–13.1)	1	(ref)
*model 3*	G1/G2	1.08	(0.51–2.29)		0.64	(0.31–1.33)	1	(ref)
	G3a	2.07	(1.12–3.84)	*	1.26	(0.68–2.33)	1	(ref)
	G3b	1.26	(0.64–2.51)		1.38	(0.71–2.71)	1	(ref)
	G4	1.82	(0.40–8.23)		2.36	(0.46–12.3)	1	(ref)
*model 4*	G1/G2	1.04	(0.48–2.25)		0.65	(0.31–1.37)	1	(ref)
	G3a	2.12	(1.13–3.98)	*	1.26	(0.68–2.34)	1	(ref)
	G3b	1.24	(0.62–2.47)		1.35	(0.69–2.65)	1	(ref)
	G4	1.77	(0.37–8.44)		2.12	(0.39–11.5)	1	(ref)
*model 5*	G1/G2	0.62	(0.23–1.68)		0.47	(0.20–1.11)	1	(ref)
	G3a	2.32	(1.09–4.94)	*	1.48	(0.72–3.06)	1	(ref)
	G3b	1.51	(0.65–3.53)		1.57	(0.69–3.56)	1	(ref)
	G4	1.90	(0.27–13.3)		1.90	(0.29–12.6)	1	(ref)
**Dependent variable: Low LVEF**								
*model 1*	G1/G2	0.85	(0.32–2.26)		0.63	(0.24–1.66)	1	(ref)
	G3a	1.62	(0.76–3.45)		1.00	(0.45–2.24)	1	(ref)
	G3b	2.86	(1.32–6.22)	**	1.79	(0.81–3.93)	1	(ref)
	G4	0.57	(0.15–2.13)		0.84	(0.20–3.50)	1	(ref)
*model 2*	G1/G2	0.86	(0.32–2.32)		0.61	(0.23–1.63)	1	(ref)
	G3a	1.51	(0.70–3.26)		1.00	(0.45–2.24)	1	(ref)
	G3b	2.81	(1.29–6.13)	**	1.77	(0.80–3.90)	1	(ref)
	G4	0.52	(0.12–2.20)		0.89	(0.19–4.12)	1	(ref)
*model 3*	G1/G2	0.71	(0.25–2.05)		0.61	(0.22–1.70)	1	(ref)
	G3a	1.49	(0.64–3.42)		0.91	(0.39–2.14)	1	(ref)
	G3b	3.76	(1.51–9.40)	**	2.48	(0.99–6.21)	1	(ref)
	G4	0.36	(0.07–1.92)		0.62	(0.11–3.51)	1	(ref)
*model 4*	G1/G2	0.65	(0.22–1.93)		0.56	(0.20–1.60)	1	(ref)
	G3a	1.43	(0.62–3.32)		0.90	(0.38–2.13)	1	(ref)
	G3b	3.96	(1.57–9.96)	**	2.51	(1.00–6.33)	1	(ref)
	G4	0.35	(0.06–1.94)		0.71	(0.12–4.20)	1	(ref)
*model 5*	G1/G2	0.51	(0.12–2.17)		0.46	(0.13–1.56)	1	(ref)
	G3a	1.50	(0.55–4.13)		1.08	(0.40–2.93)	1	(ref)
	G3b	3.22	(1.08–9.56)	*	1.73	(0.59–5.13)	1	(ref)
	G4	0.50	(0.06–4.26)		0.52	(0.07–4.13)	1	(ref)

Model 1, adjusted for sex, age and eGFR; model 2, adjusted for variables in model 1 plus corrected calcium and inorganic phosphate; model 3, adjusted for variables used in model 2 plus CRP, and diuretic use; model 4, adjusted for variables used in model 3 plus systolic blood pressure and history of hypertension; model 5, adjusted for variables used in model 3 plus intact PTH, although fewer patients were included in this analysis.

* and ** indicate p<0.05 and P<0.01, respectively;

t1, t2, and t3 indicate the lowest, middle, and highest tertile, respectively.

Among patients with CKD stage G3a and those with CKD stage G3b, the correlation coefficient (by Spearman’s test) for the association between FGF23 and α-Klotho was 0.01 (P = 0.837) and -0.03 (P = 0.370), respectively. When FGF23 (i.e., the second and third FGF23 tertiles) was included as an additional covariate in model 5, the association between the lowest α-Klotho tertile and LVH among patents with CKD stage G3a (odds ratio 2.45, 95% CI 1.12–5.33, P = 0.024) and that between the lowest α-Klotho tertile and low LVEF among patents with CKD stage G3b (odds ratio 3.21, 95% CI 1.06–9.71, P = 0.039) retained statistical significance.

When the analysis was performed using model 5 in Table 4 for patients with CKD stage either G2, G3a, G3b, or G4, the association between the lowest α-Klotho tertile and LVH (odds ratio 1.31, 95% CI 0.84–2.03, P = 0.230) or low LVEF (odds ratio 1.06, 95% CI 0.63–1.85, P = 0.849) was not significant.

### Association between FGF23/α-Klotho and LVH/low LVEF among patients with CKD stages G3-G4

We next investigated the association between FGF23 and LVH or low LVEF among the subgroup of patients with CKD who were not undergoing chronic hemodialysis (i.e., CKD stage 3a, 3b, and 4).

When the model was adjusted for the variables used in model 4 in Tables 3 or 4, the odds ratio of the highest FGF23 tertile for LVH was 1.99 (95% CI 1.27–3.10, P = 0.002) and that for low LVEF was 1.84 (95% CI 1.02–3.26, P = 0.041). Furthermore, after adjusting for the same variables, the odds ratio of the lowest α-Klotho tertile for LVH was 1.44 (95% CI 0.94–2.20, P = 0.44) and that for low LVEF was 1.52 (95% CI 0.89–2.61, P = 0.126).

### Multivariate ROC curve analysis

**Fig 5:**
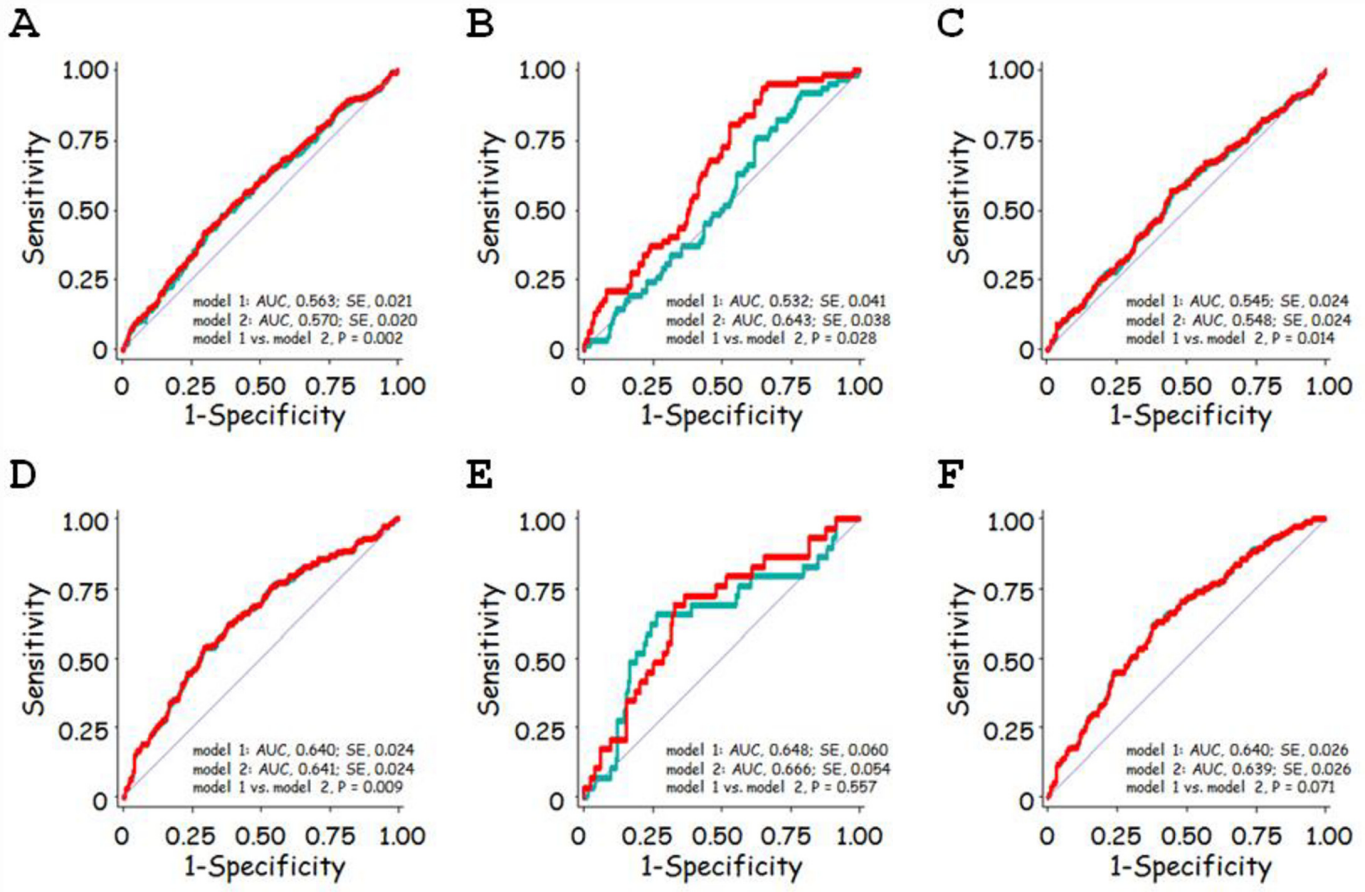
Receiver operating characteristic (ROC) analysis for the prediction of left ventricular hypertrophy (LVH) and low left ventricular ejection fraction (low LVEF). A, B, C. ROC curve for the prediction of LVH. D, E, F. ROC curve for the prediction of low LVEF. Green lines show the ROC curve to predict LVH (A, B, C) and low LVEF (D, E, F) for the combination of age, sex, and eGFR, designated model 1. Red lines show the ROC curve to predict LVH (A, B, C) and low LVEF (D, E, F) for model 1 plus FGF23, designated model 2. For the prediction of LVH, the area under the ROC curve was significantly greater in model 2 than in model 1 for patients with CKD stage G1-G4 (A), G1/G2 (B), and G3-G4 (C). For the prediction of low LVEF, the area under the ROC curve was significantly greater in model 2 than in model 1 for patients with CKD stage G1-G4 (D); however, it did not differ significantly between the two models for patients with CKD stage G1/G2 (E) or G3-G4 (F).

In ROC curve analysis for the prediction of LVH, addition of FGF23 (Fig 5A, 5B and 5C; model 2, red line) to the combination of age, sex, and eGFR (model 1, green line) significantly improved the prediction of LVH among patients with CKD stages G1-G4 (Fig 5A), G1/G2 (Fig 5B), and G3-G4 (Fig 5C), and this improvement seemed to be most prominent among those with CKD stage G1/G2.

In ROC curve analysis for the prediction of low LVEF, addition of FGF23 to the combination of age, sex, and eGFR significantly improved the prediction of LVH for patients with CKD stages G1-G4 (Fig 5D), but was not statistically significant in the subgroup of patients with CKD stage G1/G2 (Fig 5E) or G3-G4 (Fig 5F).

## Discussion

We found that serum FGF23 was significantly associated with LVH independent of sex, age, eGFR, and serum calcium and inorganic phosphate levels among patients with no CKD (i.e., CKD stage G1/G2). In addition, this association remained significant after further adjustment for intact PTH or diuretic use. In addition, it was found that serum FGF23 was significantly associated with LVEF among patients with CKD stages G1/G2 and G3a (Table 3); unexpectedly, however, the relationship was insignificant among patients with more severe renal dysfunction (CKD G3-G5). On the other hand, the relationship between α-Klotho and left ventricular mass or systolic dysfunction was not significant among patients with no CKD (stage G1/G2) (Table 4).

Similar to the current study, we previously assessed the possible relationship between FGF23 (or α-Klotho) and cardiac hypertrophy and systolic dysfunction among patients admitted to the cardiology department, although the study population was much smaller [16]. In that study, data were analyzed from a total of 100 patients that included only 30 (30%) with CKD stage G1/G2; although FGF23 was significantly correlated with LVMI and low LVEF among the whole study population, the correlations were not statistically significant among patients with no-CKD. In the current study, by increasing the number of enrolled subjects approximately nine-fold, we have demonstrated a significant association between FGF23 and LVH or low LVEF among patients with CKD stage G1/G2 after adjustment for various factors not included in the previous study, such as history of hypertension, diuretic use, and CRP.

Previously, the relationship between FGF23 and cardiac morbidity and mortality has been best demonstrated among patients with CKD. For example, Faul et al. demonstrated that, among subjects with CKD, those with higher serum FGF23 levels had a higher risk of LVH and low LVEF [7]. In addition, Mirza et al. showed that FGF23 was significantly correlated with LVMI among elderly patients with eGFR below 60 mL/min/1.73m^2^, but not among elderly patients with eGFR ≥ 60 mL/min/1.73m^2^ [9]. Furthermore, Gutierrez et al. showed that FGF23 was independently associated with LVMI and LVH among patients with CKD [8].

In a few studies, on the other hand, the relationship between FGF23 and cardiac abnormalities or cardiovascular outcome has been studied in patients without renal dysfunction or with relatively preserved renal function. For instance, Seiler et al. analyzed the data from subjects undergoing elective coronary angiography, and showed that the association between FGF23 and LVEF and that between FGF23 and LVH was significant among patients with preserved renal function (eGFR ≥ 60 mL/min/1.73m^2^) [24]. Imazu et al. showed that FGF23 was independently and negatively associated with LVEF among subjects with eGFR > 40 mL/min/1.73m^2^ [25]. In addition, Brandenburg et al. demonstrated that higher FGF23 was associated with higher cardiovascular mortality among both patients with eGFR ≥ 60 mL/min/1.73m^2^ and those with eGFR < 60 mL/min/1.73m^2^ with similar hazard ratios [26]. Furthermore, Ix et al. analyzed community-living individuals and showed that the incidence of heart failure increased with FGF23 among both individuals with CKD and those without CKD [27].

In the current study, by analyzing a relatively large study sample, we have been able to demonstrate an association, or non-association, between FGF23 and LVH and cardiac dysfunction according to CKD stage. To this end, we verified the association between FGF23 and these parameters among CKD stage G1/G2 patients in a multivariate model in which various possible confounding variables were used as covariates (Table 3, models 1–5). Furthermore, the relationship between FGF23 and LVH was significant among subjects with CKD stage G1/G2 after adjustment for CRP and diuretic use and intact PTH (model 5). It was also found that association between FGF23 and cardiac hypertrophy might be stronger among patients with CKD stage G1/G2 or CKD stage G3a than among those with CKD stage G3b (Table 3). This observation should be verified in future studies.

We found that FGF23 was not associated with LVH among patients with CKD stage G3b and that FGF23 was not associated with low LVEF among patients with CKD stage G3b and G4 in some models (Table 3); notably, however, we found a significant association between FGF23 and LVH or low LVEF among subjects with CKD stages G3-G4, which is consistent with previous reports.

Several previous studies have suggested that FGF23 may promote cardiac hypertrophy via FGF receptor 4 (FGFR4)-mediated activation of the calcineurin-NFAT signaling pathway [28,29], although to what extent circulating FGF23 reflects myocardial FGF23 in the setting of heart failure remains under discussion [30]. In the current study, the relationship between FGF23 and cardiac hypertrophy differed according to CKD stage; therefore, whether or not differences between the various CKD stages might be explained by variations in the cardiac expression of FGFR4 awaits further investigation [7,8].

In the current study, we found that lower α-Klotho levels were not associated with cardiac hypertrophy or systolic dysfunction among patients with CKD G1/G2 (Table 4); on the other hand, the lowest α-Klotho tertile was significantly associated with LVH and low LVEF among patients with CKD stage G3a and those with CKD stage G3b, respectively (Table 4). In contrast to the current observations, some previous studies did not find association between circulating α-Klotho and cardiovascular outcome. For example, Seiler et al. reported that, among 312 patients with stage G2–G4 CKD, plasma levels of Klotho did not predict an adverse outcome [31]. In addition, Buiten et al. reported that plasma Klotho was not independently associated with CVD among 127 dialysis patients [32]. Although the reason for the discrepancies between those studies and ours remains unclear, there are several possibilities. First, we assessed the association according to the different stages of CKD. In fact, when subjects with a CKD stage of G2, G3, or G4 were analyzed together in the current study, α-Klotho was not significantly associated with LVH (odds ratio 1.31, 95% CI 0.84–2.03, P = 0.230) or low LVEF (odds ratio 1.06, 95% CI 0.63–1.85, P = 0.849) by model 5 used in Table 4. Second, we examined cardiac hypertrophy and dysfunction, rather than cardiovascular outcomes. Third, the association between α-Klotho and cardiac hypertrophy or low LVEF was confounded by FGF23, which has a significant relationship with cardiomyopathy [7]. On the other hand, among patients with CKD stage G3a or G3b in the current population, the correlation between FGF23 and α-Klotho was not significant; in addition, the association between α-Klotho and LVH among patients with CKD stage 3a and that between α-Klotho and low LVEF among patients with CKD stage 3b remained statistically significant after entering FGF23 as an additional covariate in multivariate logistic regression analyses. Together these findings suggest that α-Klotho may be associated with LVH and low LVEF in a FGF23-independent manner in some limited CKD conditions.

What might be the possible mechanism, if present at all, by which circulating α-Klotho exerts a direct effect on cardiac cells? Xie et al. demonstrated that cardiac hypertrophy and dysfunction were aggravated in response to the induction of renal failure in heterozygous klotho deficient mice; this aggravation was independent of hypertension or FGF23, and was ameliorated by *klotho* gene delivery [33]. Mechanism by which Klotho deficiency aggravated cardiomyopathy in the setting of renal dysfunction is unknown, but it might be related to the observation that soluble Klotho inhibits stress-induced cardiac TRPC6 expression and activation of NFAT [34], which in turn may induce fetal gene expression and pathologic cardiac hypertrophy and remodeling [35]. It has also been shown that myocardial FGF23/FGFR4 expression is associated with cardiac hypertrophy among patients with end-stage renal disease [29]. Whether cardiac FGF23 is increased among non-CKD patients with cardiac hypertrophy should be investigated in future studies.

There are several limitations to the current study. First, owing to the cross-sectional nature of the study, we cannot conclude whether the relationship between FGF23 and cardiac hypertrophy or systolic dysfunction was causal or resultant. Second, we did not take into account 25(OH)D3 or 1,25(OH)_2_D3, which may have a relationship with cardiac parameters [16]. On the other hand, a strength of the current study is that by including 234 patients who had CKD stage G1 or G2, we could analyze whether serum FGF23 value is useful biomarker for cardiac hypertrophy and left ventricular systolic dysfunction among patients who have no or only minor renal dysfunction. In addition, by increasing overall study sample, we could statistically analyze the association, or non-association, between FGF23/α-Klotho and LVH/low LVEF among the patients with various subcategories (i.e., CKD stage G3a, G3b, and G4) of renal dysfunction. Furthermore, in the logistic regression model, the significant association between FGF23 and cardiac hypertrophy was retained after adjusting for not only serum levels of calcium and inorganic phosphate but also use of diuretic drugs, which have been shown to potently alter serum FGF23 [36].

It should be noted that two-types of ELISA are available for the determination of human FGF23: an assay for intact FGF23 that detects only full-length FGF23, and one for C-terminal FGF23. In the current study, we used the ELISA for full-length FGF23. Some previous studies have used the full-length assay, and thus measured serum levels of intact FGF23 [9,10], whereas others have used the C-terminal FGF23 assay (Immutopics) [7,8,11]. C-terminal FGF23 concentration has been reported to have not only a strong linear correlation with intact FGF23 concentration [8], but also an association with adverse cardiovascular and renal events [37,38]. On the other hand, recent studies suggested an increase in C-terminal FGF23 may precede an increase in intact FGF23 in the relatively early phase of CKD [39] and that cleavage of FGF23 might be a major determinant for FGF23 activity in various diseases [40,41,42]; therefore, serum levels of intact FGF23 should be assessed, especially among populations including patients without severe renal dysfunction, such as the current study sample.

In summary, by analyzing data from 903 cardiology patients who had various degrees of renal dysfunction, we found that FGF23 levels were associated positively and negatively with, respectively, LVH and low LVEF independent of age, sex, eGFR, corrected calcium, CRP, diuretic use, systolic blood pressure, and history of hypertension not only among patients with CKD (CKD stage G3a/G3b/G4) but also those without (CKD stage G1/G2). The association between serum α-Klotho and LVH and low LVEF was significant among patients with CKD stage G3b and G3a, respectively, after adjustment for age, sex, and eGFR. Whether modulating FGF23 levels might have an impact on cardiac remodeling and functional impairment irrespective of the CKD status awaits further investigation.
